# Trends of liver cancer and its major risk factors in Korea

**DOI:** 10.4178/epih/e2015016

**Published:** 2015-03-11

**Authors:** Eun-young Lee, Tran Thi Xuan Mai, Yoonjung Chang, Moran Ki

**Affiliations:** Department of Cancer Control and Policy, Graduate School of Cancer Science and Policy, National Cancer Center, Goyang, Korea

**Keywords:** Liver cancer, Hepatitis B virus, Hepatitis C virus, Alcoholic liver cirrhosis

## Abstract

The Republic of Korea (hereafter Korea) is one of the countries with high incidence of liver cancer and there is great difference in incidence of liver cancer between male and female. We investigated the sex-specific trends of three major risk factors of liver cancer, which are hepatitis B virus(HBV) infection, hepatitis C virus(HCV) infection, and alcoholic liver cirrhosis. The incidence of liver cancer was obtained from the Cancer Registration Statistics of the National Cancer Center of Korea. Hepatitis B surface antigen (HBsAg) seropositivity was based on data from the 2011 Korea National Health and Nutrition Examination Survey. Disease statistics from the Health Insurance Review and Assessment Service of Korea were used to evaluate trends in HCV infection and alcoholic liver cirrhosis. The prevalence of these risk factors were compared with the incidence of liver cancer. Males had a three to four times higher incidence of liver cancer than females did from 1999 to 2011. This gap between the incidence for males and females increased with age and males aged 50 to 59 showed a five times higher incidence than females of the same age did. In general, HBsAg seropositivity decreased from 1998 to 2011. The prevalence of HCV infections was 96.2 and 90.3 per 100,000 females and males, respectively in 2013. The prevalences of HCV infections from 2009 to 2013 did not substantially differ. The annual average prevalence of alcoholic liver cirrhosis from 2009 to 2013 was 77.22 and 8.90 per 100,000 males and females, respectively; the prevalence among males was 8.7 times higher than that among females. The prevalence rapidly increased with age in males, and males aged 60 to 69 peaked with a 19.2 times higher prevalence than that among females of the same age group. We found that the incidence of alcoholic liver cirrhosis, a major risk factor of liver cancer, exhibited a trend similar to that of liver cancer incidence in males, and this trend also differed remarkably by sex.

## INTRODUCTION

### Incidence, prevalence, and mortality of liver cancer

Liver cancer is one of the most common cancers and the second most common cause of death from cancer worldwide [1], and liver cancer mortality is especially high in Asian and African countries [2]. Moreover, the incidence of liver cancer in males and females differs significantly worldwide. The 5-year prevalence of liver cancer for both sexes was estimated as 3.7% [2]. Overall, developing countries experience the greatest burden of liver cancer, approximately 85% of the all cases occurred in developing countries [1,3].

The Republic of Korea (hereafter Korea) has a high endemic population of patients with chronic hepatitis B virus (HBV) infections, which is one of the major risk factors of liver cancer [3,4]. In 2011, liver cancer accounted for 18.3% of all cancer deaths in Korean males and 10.2% of all cancer deaths in Korean females. Although the 5-year relative survival of liver cancer in Korea has demonstrated an increasing trend over the last two decades, it remains one of the lowest survival rates at only 26.7% from 2006 to 2010 [4]. According to results published by GLOBOCAN 2012, the incidence of liver cancer in Korea is the sixth highest in the world and the fourth highest among all Asian countries [2].

### Major risk factors of liver cancer in Korea

In the Korean National Cancer Screening Program, people infected with the HBV, or hepatitis C virus (HCV) as well as those with liver cirrhosis are categorized as having a high risk of developing liver cancer, and these people are main targets of the liver cancer screening program [5]. Another risk factor, which is generally pointed out, is excessive alcohol consumption. According to the National Cancer Information Center of Korea, liver cancer etiology was attributed to an HBV infection, HCV infection, or harmful alcohol consumption in 74%, 9%, and 7% of these patients, respectively.

We aimed to compare the recent trends of infections with HBV and HCV, as well as the trend of alcoholic liver cirrhosis in Korea to investigate whether differences in these trends by sex are related to difference in the sex-specific incidence of liver cancer in Korea.

## MATERIALS AND METHODS

### Incidence of liver cancer in Korea

The annual incidence of liver cancer in Korea was calculated by sex from 1999 to 2011 using data from the Korea Central Cancer Registry [6].

In addition, the age-specific incidence of liver cancer in Korea was calculated using 2011 data from the Cancer Registry statistics, which collected data on the age-specific crude incidence of liver cancer. Liver cancer is categorized as C22 (malignant neoplasm of the liver and intrahepatic bile ducts according to Korean Standard Classification of Diseases (KCD) [7].

### Risk factors

#### Hepatitis B Virus

Hepatitis B surface antigen (HBsAg) seropositivity was calculated as the percentage of individuals who tested positive for HBsAg. The percentage of HBsAg seropositivite individuals from 1998 to 2011 and the age-specific percentage of HBsAg seropositive individuals in 2011 were adapted from data provided by the 2011 Korea National Health Nutrition Examination Survey (KNHANES) [8]. The surveyed population in the KNHANES was over 10 years old, and the percentages of seropositive individuals were calculated using age-standardized weights based on the total estimated population of Korea in 2005.

#### Hepatitis C Virus

The prevalence of HCV infection from 2009 to 2013 was calculated as the total number of patients with an HCV infection as reported by the Health Insurance Review and Assessment Service of Korea (HIRA) [9]. In addition, data maintained by the Ministry of Security and Public Administration on the total population of registered residents in Korea were used for prevalence estimations [10]. The number of patients with an HCV infection in the present study includes both the number of acute and chronic HCV infection patients, which are coded as B171 and B182, respectively, according to the KCD. To calculate the annual prevalence, the numbers of patients obtained from these datasets were divided by the total population for each year.

To calculate the age-specific prevalence, the data from 2011 were chosen because other factors that might be potentially related to this age-specific data were available in the 2011 data. The number of patients with acute or chronic HCV infections in the 2011 HIRA data was divided by the age-specific population that was provided by the Ministry of Security and Public Administration.

#### Alcoholic liver cirrhosis

The prevalence of alcoholic liver cirrhosis was calculated from 2009 to 2013 (code K703 in the KCD), using the HIRA data as well as the annual Ministry of Security and Public Administration data [9,10]. The prevalence of alcoholic liver cirrhosis was calculated by dividing the number of alcoholic liver cirrhosis patients by the annual population for each sex separately.

In addition, the age-specific prevalence in 2011 was calculated by dividing the number of patients with alcoholic liver cirrhosis by the total age-specific population.

## RESULTS

### Incidence of liver cancer by year

**Figure 1.**
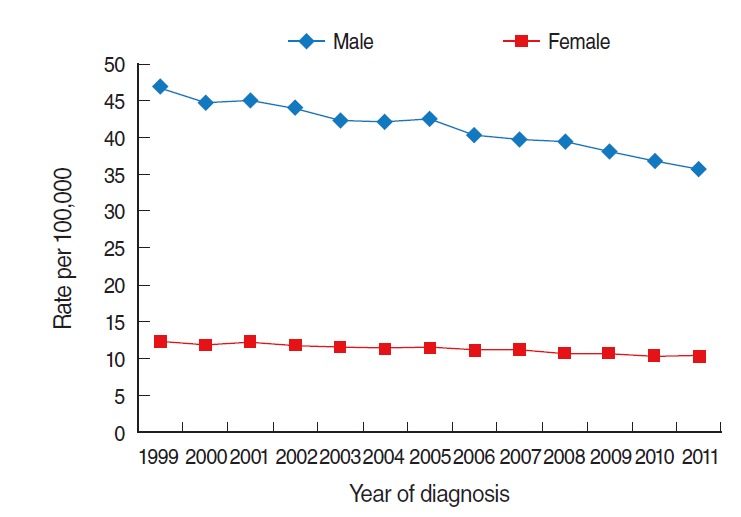
Age-standardized incidence of liver cancer in Korea by year, 1999-2011 (standard population: Korean population in 2000). From Jung KW, et al. Cancer Res Treat 2014;46:109-123 [6].

The incidence of liver cancer among males was three to four times higher than that among females from 1999 to 2011. In 2011, the incidences among males and females were 35.6 and 10.3 per 100,000 persons, respectively. In both sexes, the incidences demonstrated a decreasing trend over time (Figure 1).

**Figure 2.**
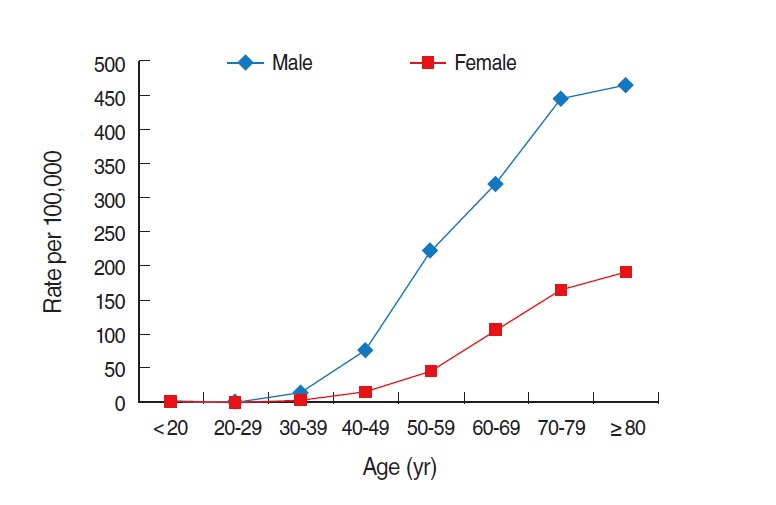
Age-specific crude incidence of liver cancer in Korea by sex in 2011. From Korean Statistical Information Service. Number of cancer patients, relative frequency, crude rate, age-adjusted incidence by cancer site and sex from 1999 to 2011 [7].

Figure 2 illustrates the annual trends in the age-specific incidence of liver cancer by sex in Korea. Although the incidence among males and females increased with age, males exhibited a sharper increase over time. The biggest difference between the incidences for males and females appeared in those aged 50 to 59 years old with an incidence of 223.2 and 44.5 per 100,000 males and females, respectively. Thus, males showed five times higher incidence of liver cancer than females did (Figure 2).

### Risk Factors

#### Hepatitis B virus

**Figure 3.**
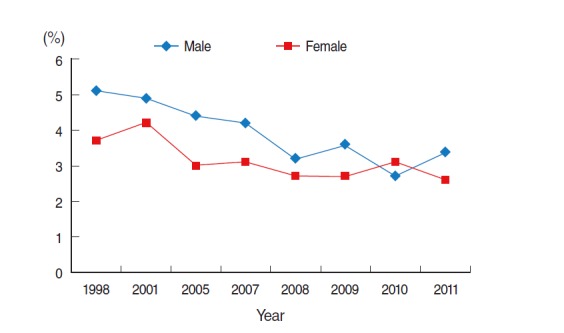
Trend of hepatitis B surface antigen (HBsAg) seropositivity in Korea 1998 to 2011. From Korea Centers for Disease Control and Prevention. Korea health statistics 2011: Korea National Health and Nutrition Examination Survey. Cheongju: Korea Centers for Disease Control and Prevention; 2012 [8]. HBsAg seropositivity, percentage of individuals who have tested positive to HBsAg (those who are 10 years old and over).

The prevalence of HBsAg seropositivity tended to decrease overall in both males and females. In general, the prevalence among males was slightly higher than that among females. The average annual prevalence of HBsAg seropositivity was 3.94 and 3.13% in males and females, respectively. This prevalence is likely to have resulted after the initiation of the National Vaccination Program, which started in 1995. In addition, in 2002, the Hepatitis B Perinatal Transmission Prevention Program started in Korea (Figure 3).

**Figure 4.**
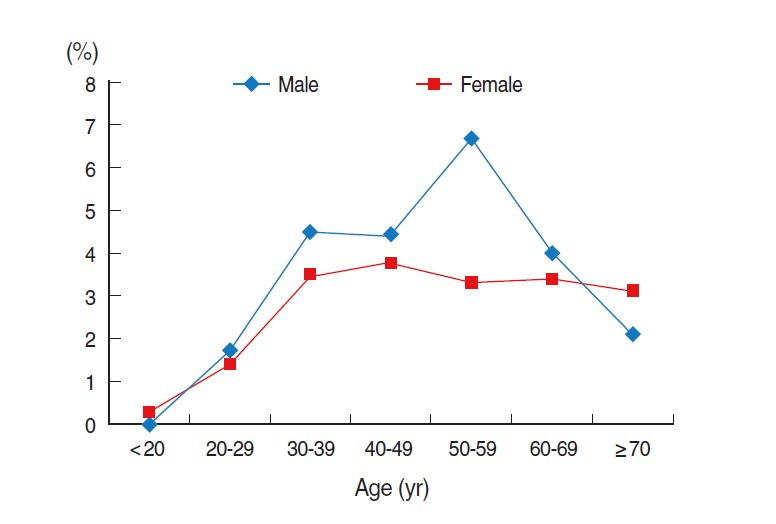
Age-specific hepatitis B surface antigen seropositivity in Korea in 2011. From Korea Centers for Disease Control and Prevention. Korea health statistics 2011: Korea National Health and Nutrition Examination Survey. Cheongju: Korea Centers for Disease Control and Prevention; 2012 [8].

Generally, more males were HBsAg seropositive than females were. However, among those under 20 years old and over 70 years old, females had a higher prevalence of HBsAg seropositivity than males did. Furthermore, among those aged 50 to 59, the prevalence of HBsAg seropositivity was approximately two times higher in males compared to females (Figure 4).

#### Hepatitis C virus

**Figure 5.**
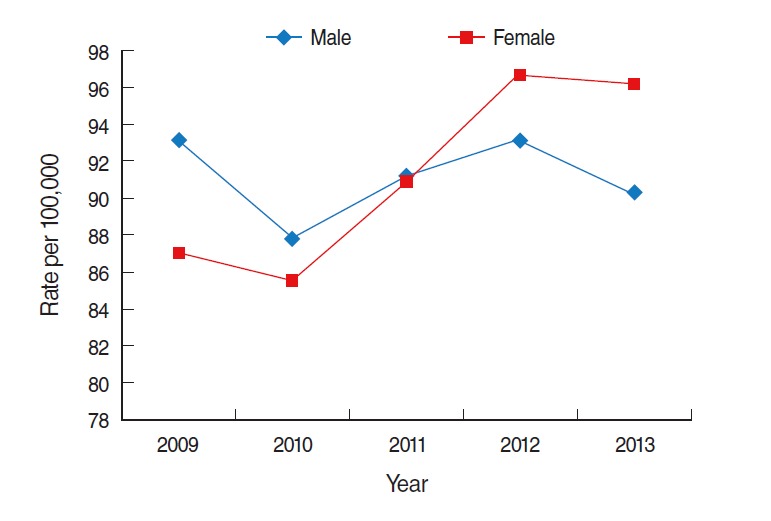
Trend of prevalence of hepatitis C virus infection in Korea from 2009 to 2013. From Health Insurance Review and Assessment Service. Disease statistics [9]; Ministry of Security and Public Administration. Registered population statistics from 2009-2013 [10].

The average annual prevalences of HCV infection were 93 and 89 per 100,000 males and females, respectively. This rate tended to fluctuate slightly from year-to-year (Figure 5).

**Figure 6.**
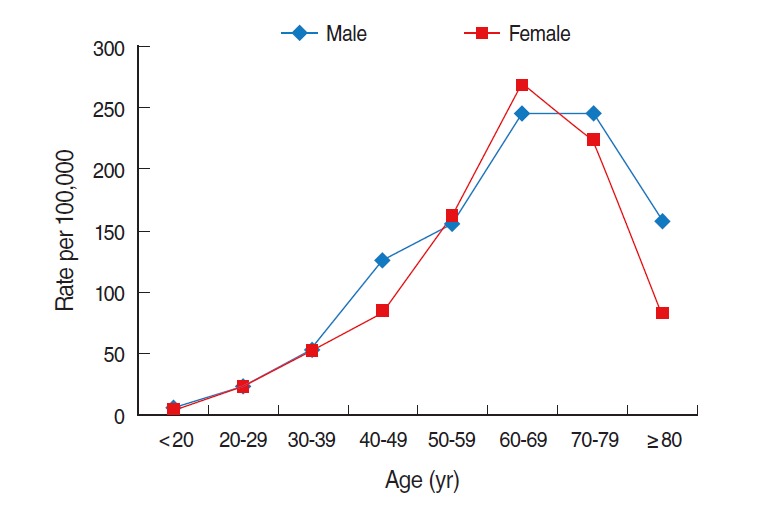
Age-specific prevalence of hepatitis C virus infection by age in Korea in 2011. From Health Insurance Review and Assessment Service. Disease statistics [9]; Ministry of Security and Public Administration. Registered population statistics from 2009-2013 [10].

The prevalence of HCV infection by age showed a similar trend in both sexes: it increased with increasing age and peaked among those aged 60 to 69 for both males and females (Figure 6).

#### Alcoholic liver cirrhosis

**Figure 7.**
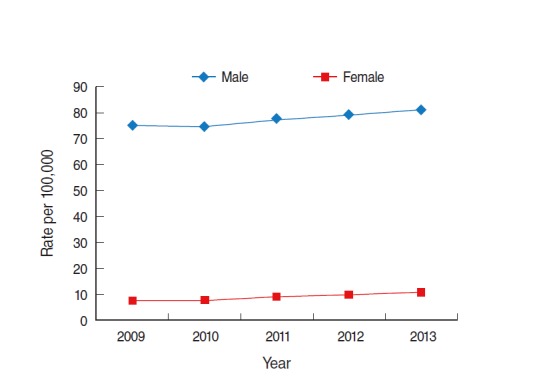
Trend of prevalence of alcoholic liver cirrhosis in Korea from 2009 to 2013. From Health Insurance Review and Assessment Service. Disease statistics [9]; Ministry of Security and Public Administration. Registered population statistics from 2009-2013 [10].

In both sexes, the prevalence of alcoholic liver cirrhosis constantly increased each year. However, the prevalence among males was much higher than that among females for every year. For example, in 2009 and 2013, the prevalence among males was approximately 10 times and 8 times higher than that among females was, respectively. On average, the annual prevalence among males was 8.7 times higher than that among females was (Figure 7).

**Figure 8.**
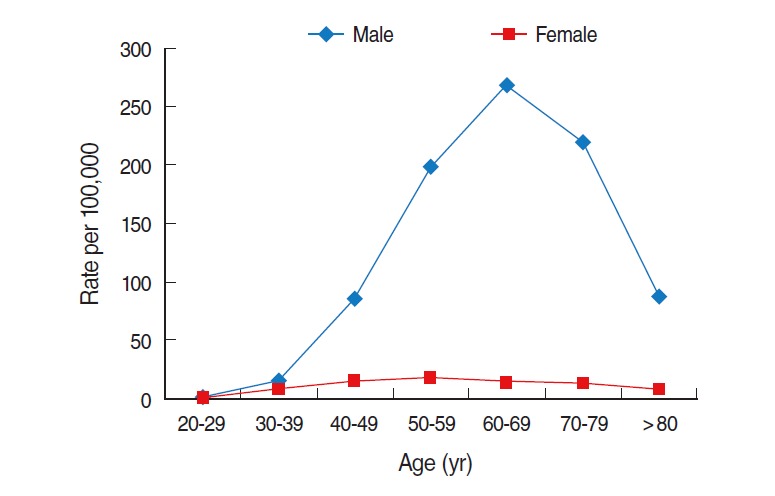
Age-specific prevalence of alcoholic liver cirrhosis in Korea in 2011. From Health Insurance Review and Assessment Service. Disease statistics [9]; Ministry of Security and Public Administration. Registered population statistics from 2009-2013 [10].

For the age-specific prevalence, the prevalence among females remained under 20 per 100,000 females in all age groups, and this prevalence peaked in the 50 to 59 years old age group. However, the prevalence among males increased sharply with age, and it peaked in the 60 to 69 years old age group with the overall highest prevalence of 268.3 per 100,000 males. The prevalence among males older than 69 years decreased with age, but the rate remained much higher than that among their female counterparts. In the 60 to 69 years old age group, males had a prevalence that was 19 times higher than that of females in the same age group (Figure 8).

## DISCUSSION

### Liver cancer incidence trends

The age-specific incidence of liver cancer observed in Korean males follows a trend that is similar to that in other Asian countries including China, Japan, the Philippines, Singapore, and Thailand. The incidence tends to increase gradually with age, and therefore those aged >80 years possess the highest incidence. Furthermore, in China and Japan, the gap in the incidence of liver cancer between males and females tends to become more apparent with increasing age [2].

We observed a modest decrease in the incidence of liver cancer in Korea in both sexes from 1999 to 2011. According to GLOBOCAN 2012, this trend in Korea is similar to that in Singapore and China. In European countries, liver cancer incidence among males has remained relatively stable for a long period (since 1975 until now), and for European females, it has slightly increased since 1975. The incidence of liver cancer in the US has exhibited an opposite trend compared with the trend in Korea. In the US, recent statistics have demonstrated that liver cancer has gradually increased in both sexes [2].

**Figure 9.**
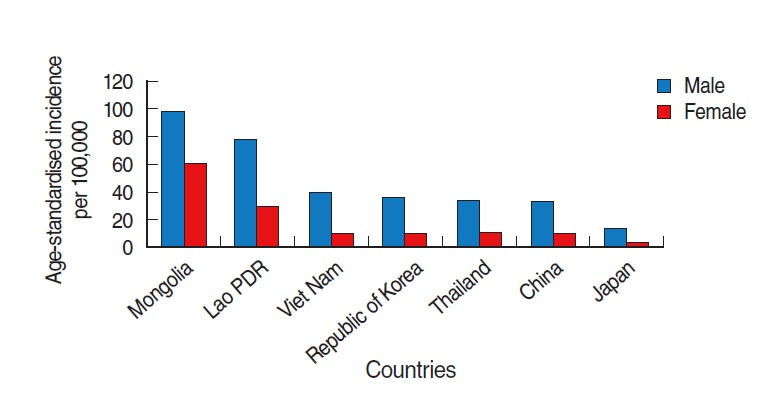
Age-standardised incidence rate of liver cancer in some Asian countries. From International Agency for Research on Cancer. GLOBOCAN 2012: estimated cancer incidence, mortality and prevalence worldwide in 2012 [2].

We also observed a substantial gap in the incidence rate of liver cancer between males and females in Korea. Other Asian countries with a high incidence of liver cancer also share a similar pattern. The sex ratio of the incidences among males to females was found to be greater than 3.0 in some countries including Vietnam (3.6), Thailand (3.1), China (3.1), and Japan (3.1). Males tend to have a higher liver cancer incidence than females in almost all populations, and the sex ratio usually fluctuates between 2:1 and 4:1 (Figure 9) [11].

### Risk factors of liver cancer in Korea

#### Hepatitis B virus infection

HBV infection has been considered a major risk factor in the development of liver cancer. In Korea, the prevalence of HBsAg seropositivity showed an overall decreasing trend from 1998 to 2011 because of a national vaccination program that was initiated in 1995. Liver cancer caused by HBV infection is expected to decrease considering the positive effect of the HBV vaccination [12]. Among children under 10 years old, the prevalence of HBsAg seropositivity remarkably decreased to 0.2% in 2007 [13]. The difference between the average annual HBsAg seropositivity from 1998 to 2011 for males and females was 0.81%.

#### Hepatitis C virus infection

For HCV infections, no vaccination is available; however, the incidence is not relatively high. Nevertheless, more than 80% of those infected with the HCV are likely to develop chronic HCV infections that can easily advance into liver cancer in some individuals. The cumulative incidence of liver cirrhosis that leads to liver cancer has been reported to be higher among those with an HCV infection than that among those with an HBV infection is. Moreover, with time, the burden rate of developing liver cancer was found to be higher among those with HCV infections than that among those with HBV infections was [14]. Both sexes showed a similar trend for the prevalence of HCV infection by year and age group.

#### Alcoholic liver cirrhosis

Korea has a very high rate of alcohol consumption, and as of 2010, approximately 10.3% of all Korean males had an alcohol use disorder and/or alcohol dependence. Among Korean females, this percentage was 2.2%.The average estimate in the World Health Organization Western Pacific Region was 4.6%. In the same year, the total alcohol consumption per capita was 37.6 L in Korean males and 11.5 L in Korean females. Moreover, the prevalence of heavy episodic drinking in Korea was markedly different between males and females at 12.1% for males and 0.1% for females [15].

For the causes of liver cirrhosis in Korea, HBV infections and alcohol use account for 64.9 and 18.6% of all liver cirrhosis cases, respectively. According to an epidemiological survey, the prevalence of alcoholic liver disease is correlated with the per capita alcohol consumption, and the risk of alcoholic hepatitis and liver cirrhosis increased with increasing alcohol consumption [16].

In the present study, the incidence of liver cancer and prevalence of alcoholic liver cirrhosis demonstrated similar trends. Both of these indices differed greatly between males and females, and males showed much higher rates than females did. The difference in alcohol consumption between males and females might explain the big gap between the male and female liver cancer incidence rates in Korea. Currently, the Ministry of Health and Welfare of Korea is investigating several measures that might regulate liquor advertising and limit the number of places alcohol can be purchased [17].

Previous studies found significant associations between heavy alcohol consumption and the risk of hepatocellular carcinoma in HBV and HCV patients. One study from Taiwan concluded that excessive alcohol consumption dramatically increased the risk of hepatocellular carcinoma in patients with HBV-related cirrhosis [18]. A similar result was found in a study on a US population, which stated that moderate and excessive alcohol consumption increased the risk of liver-related mortality in HCV patients [19].

#### Limitations

Our study has several limitations. First, secondary data was used for our analysis. However, the similar trends found between incidence of liver cancer and prevalence of alcoholic liver cirrhosis still can suggest that alcohol might play a critical role regarding big difference in the incidence of liver cancer between males and females. Further studies are needed.

Second, the numbers of patients with HCV infections and alcoholic liver cirrhosis were calculated using the HIRA dataset of Korea. These disease statistics were calculated using data on medical care expenses requested from hospitals and clinics. In this data, cases classified as main disease only were included and secondary cases or the patients who did not visit hospital were not included.

Last, we only evaluated three main risk factors of liver cancer in Korea. However, several other potential factors may have caused the gender disparity in the incidence of liver cancer. For example, one controversial factor is the effect of tobacco consumption on liver cancer incidence. Further studies on the causes of the gender disparity in liver cancer are needed.

Most noticeable characteristic in incidence of liver cancer in Korea is that male continuously showed much higher incidence than female. In this research the similarity was found in the incidence of alcoholic liver cirrhosis among main risk factors of liver cancer in Korea. It opens the possibility that harmful use of alcohol of male might have led to much higher incidence of liver cancer of them. Further study is needed for the verification.
